# Identification of Candidate Chemosensory Receptors in the Antennae of the Variegated Cutworm, *Peridroma saucia* Hübner, Based on a Transcriptome Analysis

**DOI:** 10.3389/fphys.2020.00039

**Published:** 2020-01-31

**Authors:** Ya-Lan Sun, Jun-Feng Dong, Nan Gu, Shao-Li Wang

**Affiliations:** ^1^Forestry College, Henan University of Science and Technology, Luoyang, China; ^2^Department of Plant Protection, Institute of Vegetables and Flowers, Chinese Academy of Agricultural Sciences, Beijing, China

**Keywords:** odorant receptor, gustatory receptor, ionotropic receptor, antennae, transcriptome, *Peridroma saucia*

## Abstract

Insect chemoreception, including olfaction and gustation, involves several families of genes, including odorant receptors (ORs), ionotropic receptors (IRs), and gustatory receptors (GRs). The variegated cutworm *Peridroma saucia* Hübner (Lepidoptera: Noctuidae) is a worldwide agricultural pest that causes serious damage to many crops. To identify such olfactory and gustatory receptors in *P. saucia*, we performed a systematic analysis of the antennal transcriptome of adult *P. saucia* through Illumina sequencing. A total of 103 candidate chemosensory receptor genes were identified, including 63 putative ORs, 10 GRs, 24 IRs, and 6 ionotropic glutamate receptors (iGluRs). Phylogenetic relationships of these genes with those from other species were predicted, and specific chemosensory receptor genes were analyzed, including ORco, pheromone receptors (PRs), sugar receptors, CO_2_ receptors, and IR co-receptors. RT-qPCR analyses of these annotated genes revealed that 6 PRs were predominantly expressed in male antennae; 3 ORs, 1 GR, 2 IRs, and 2 iGluRs had higher expression levels in male than in female antennae; and 14 ORs, 1 GR, and 3 IRs had higher expression levels in female than in male antennae. This research increases the understanding of olfactory and gustatory systems in the antennae of *P*. *saucia* and facilitates the discovery of novel strategies for controlling this pest.

## Introduction

Insects rely on chemoreception to recognize and discriminate chemical cues in the external environment in order to mate, oviposit, locate hosts, and avoid predators (Hansson and Stensmyr, 2011; Leal, 2013). Insect chemosensation is mainly mediated by three families of chemosensory receptor genes including odorant receptors (ORs) (Clyne et al., 1999; Vosshall et al., 1999), gustatory receptors (GRs) (Clyne et al., 2000), and ionotropic receptors (IRs) (Benton et al., 2009). These genes are usually expressed in the primary olfactory appendages, the antennae.

Insect ORs were first identified in the *Drosophila melanogaster* genome (Gao and Chess, 1999). ORs are seven-transmembrane proteins with an intracellular N-terminus and extracellular C-terminus, which is opposite to the topology of the G protein-coupled ORs in vertebrates. It transpires that insect odorant receptors are heterodimers composed by one tuning OR subunit and one conserved odorant receptor co-receptor (ORco), acting as non-selective ligand-gated ion channels (Sato et al., 2008; Wicher et al., 2008). Genes in the OR family differ greatly among insect species (except for ORco), both in sequence and in the total number of ORs expressed (Engsontia et al., 2008; Zhou et al., 2015).

Insect GRs, which were also first identified in *D. melanogaster*, are mainly expressed in taste organs and are associated with contact chemoreception (Clyne et al., 2000; Scott et al., 2001). Like the OR family, the GR family includes many related members with sequences and numbers that vary greatly across species, except that carbon dioxide (CO_2_) receptors and sugar receptors, which are often expressed in antennae, are conserved among insects (Kwon et al., 2007; Sato et al., 2011).

Ionotropic receptors are related to a subfamily of ancient and highly conserved ionotropic glutamate receptors (iGluRs) (Benton et al., 2009). Genes in the IR family, which have been well studied in *D. melanogaster*, and play key roles in sensing different odorants, acids, salts, aldehyde, ammonia, temperature, and humidity (Chen et al., 2015; Enjin et al., 2016; Knecht et al., 2016; Frank et al., 2017). Based on amino acid sequences and expression patterns, the IR family in Lepidoptera can be divided into three subgroups. The first subgroup, “antennal IRs”, comprises proteins that are specifically expressed in insect antennae involved in olfaction, gustation, thermosensation and hygrosensation (Croset et al., 2010). The majority of the IRs belong to the second IR subgroup, “divergent IRs”. The copy numbers of these receptors are highly variable across species, and they appear to be absent from antennae, and function in gustation (Croset et al., 2010). A third group of IRs occurs in moths and butterflies, and was recently proposed to be Lepidoptera-specific (Liu et al., 2018). In addition, several co-receptor lineages (including IR8a, IR25a, and IR76b) have also been reported. The functional IR in this family is a heteromeric complex composed of at least one specific ligand-detecting IR and a IR co-receptor (Abuin et al., 2011; Fleischer et al., 2018).

The variegated cutworm *Peridroma saucia* Hübner (Lepidoptera, Noctuidae) is highly polyphagous, attacking more than 121 plant species including tobacco, corn, potato, wheat, and sorghum (Rings et al., 1976). *Peridroma saucia* was first recorded in Europe in 1790 and then caused serious outbreaks in many countries throughout the Americas in 1841 (Capinera et al., 1988). It has been damaging crops in North America and Europe for at least 40 years (Rings et al., 1976; Inomata et al., 2002; Choi et al., 2009). Since the 1970s, it has spread as an invasive pest in Japan and Korea and gradually become an important agricultural pest worldwide (Struble et al., 1976; Simonet et al., 1981; Willson et al., 1981). In China, the first outbreak of *P. saucia* occurred in Sichuan Province in 1981 (Kuang, 1985). This pest has been reported in more than 12 provinces in China (Li et al., 2007; Guo et al., 2010; Xuan et al., 2012). In 2017, we found a serious outbreak of *P. saucia* in a soybean field in the suburbs of Luoyang, Henan Province (personal observation). To date, studies on *P. saucia* chemoreception are limited to measurements of the attractiveness of female sex pheromone gland components to males. Field trapping studies found that mixtures of Z11-16: OAc (major component) and Z9-14: OAc (minor component) at the ratio of 3:1 could attract a large number of males in a vegetable field in Tokyo (Inomata et al., 2002), and similar findings have been reported in South Korea (Choi et al., 2009). However, the chemosensory receptors responsible for the sensing of odors in the external environment (such as sex pheromones and host plant volatiles) by *P. saucia* remain to be identified.

In this study, we used the Illumina sequencing platform to sequence and analyze the antennal transcriptome of male and female *P. saucia*. We found a total of 103 candidate chemosensory receptor genes including 63 ORs, 10 GRs, 24 IRs, and 6 iGluRs. Expression profiles of these genes in male and female antennae were also investigated using real-time quantitative-PCR (RT-qPCR). We also analyzed the evolutionary relationships of the identified genes with the chemoreceptors of other insect species. The results provide a foundation for future functional characterization of the chemoreceptor genes in *P. saucia*.

## Materials and Methods

### Insects Rearing

A colony of adult *P. saucia* was collected from Luoyang, Henan Province, China. Forty adults in a sex ratio of 1:1 were kept in a cage (25 cm in diameter, 40 cm in length) for mating and oviposition. The larvae that hatched from the eggs were kept in a rearing room (27 ± 1°C, with 70 ± 5% relative humidity and a 16-h L/8-h D cycle) and were fed an artificial diet, the main components of which were wheat germ and soybean flour. Pupae were sexed, and male and female pupae were placed in separate cages for eclosion; the adults were given a 10% (V: V) honey solution.

### Tissue Collection and RNA Extraction

For transcriptome analysis and RT-qPCR, 100 male and 100 female antennae were collected separately from *P. saucia* on the 3rd-day after eclosion. These samples were immediately frozen in liquid nitrogen and stored at –80°C before RNA extraction. Total RNA was extracted following the manufacturer’s instructions for the RNeasy Plus Mini Kit (Qiagen, Venlo, Netherlands). The quality and concentration of the RNA were checked by 1.5% agarose gel electrophoresis and with a Nano Drop 2000 spectrophotometer (Nano-Drop Products, Wilmington, DE, United States).

### Sequencing and Assembly

cDNA library construction and Illumina sequencing of the samples were performed at Biomarker Technologies (Shunyi, Beijing, China). A 5-mg quantity of total RNA from the female or male antennae (three biological replications) was used for the synthesis of duplex-specific nuclease-normalized cDNA. The cDNA libraries were prepared using Illumina’s sample preparation instructions (Illumina, San Diego, CA, United States). The cDNA libraries were then sequenced to obtain 100-bp paired-end reads using the Illumina HiSeq 2000 platform. Adaptor sequences were removed, and low quality reads were trimmed using Trimmomatic. Transcriptome *de novo* assembly was carried out with the assembly program Trinityrnaseu-r2013-02-25. The Trinity outputs were clustered by TGICL and were finally capped using Cap3 to produce the genes. Male- and female-derived reads were combined into the assembly. Consensus cluster sequences and singletons formed the final gene dataset.

### Functional Annotation and Identification of Chemosensory Receptors

Gene annotation was performed by BLAST searching against the non-redundant (NR) database at NCBI^1^, Swiss-Prot^2^, cluster of orthologous groups of proteins (COG), protein family (Pfam) database, and gene ontology (GO) databases with an *E*-value cut-off of 1e-5 to retrieve proteins with the highest sequence similarity along with their putative functional annotations (Altschul et al., 1997; Ashburner et al., 2000; Deng et al., 2006). The BLAST results were then imported into KOBAS2.0 software^3^ for Kyoto encyclopedia of genes and genomes (KEGG) annotation (Kanehisa, 2004; Xie et al., 2011). Candidate genes encoding putative ORs, GRs, or IRs/iGluRs were identified, and the annotation results were rechecked using BLASTx in protein databases at NCBI. Open reading frames (ORFs) of candidate chemoreceptor genes were then predicted using ORFfinder^4^, and were translated into amino acid sequences in Translate at ExPASy^5^. The transmembrane domains (TMDs) of candidate ORs, GRs, and IRs/iGluRs were predicted using TMHMM server version 2.0^6^.

The expression levels of these genes were estimated using the FPKM (fragments per kilobase of transcript per million fragments mapped) method. The average FPKM value of three biological replications of each sample was calculated.

### Phylogenetic Analysis

Odorant receptors, GR, and IR/iGluR phylogenetic trees were built based on amino acid sequences from the datasets of insect species including *P. saucia* (this study), *Helicoverpa armigera*, *Bombyx mori*, and *D. melanogaster*. Amino acid sequences were first aligned using the program ClustalX (Thompson et al., 1994). Maximum- likelihood trees were constructed using the MEGA 7.0 program (Kumar et al., 2016). Bootstrap analyses of 1000 replicates were used to assess the reliability of nodes in the phylogenetic tree. The evolutionary distances were computed using the JTT matrix-based method (Jones et al., 1992). All ambiguous positions were removed for each sequence pair. Phylogenetic trees were visualized with Figtree^7^.

### RT-qPCR

RT-qPCR was performed to evaluate the relative expression levels of OR, GR, and IR/iGluR genes in male and female antennae of *P. saucia*. Total RNA was extracted from each sample and reverse-transcribed into first-strand cDNA. The newly synthesized cDNA was used as a template for RT-qPCR. Operations were then carried out following the manufacturer’s instructions for SYBR Premix ExTaq II (Tli RNaseH Plus, Takara, Dalian, China) using the StepOne Plus Real-time PCR System (Applied Biosystems, Foster City, CA, United States). The RT-qPCR conditions were as follows: one cycle of 95°C for 3 min; 40 cycles of 95°C for 10 s and 60°C for 30 s; followed by 95°C for 1 min and 55°C for 1 min. The *P. saucia* actin gene was chosen as the endogenous control and was used for normalizing target gene expression. Expression levels of chemosensory receptor genes were calculated using the 2^–ΔCt^ method (Schmittgen and Livak, 2008). Each reaction was performed in triplicate for each of three biological replicates. All primers used in the experiment (including the reference gene) are listed in Supplementary Table S1. Before RT-qPCR analysis, preliminary experiments were carried out in which five random PCR products were sequenced to confirm that they were our targets. Data were analyzed by Student’s *t*-tests, and all figures were made in GraphPad Prism 6 (GraphPad Software Inc., San Diego, CA, United States). The level of significance was set at *P* < 0.05.

## Results

### Antennal Transcriptome Sequencing and Sequence Assembly

The RNA extracted from the female and male antennae of *P. saucia* was sequenced using the Illumina HiSeq 2000 platform. A total of 83.28 million (mean length 98 bp) and 77.21 million (mean length 97 bp) clean reads were produced from female and male samples, respectively. The percentage of Q30 bases in each sample was ≥89.17% (Supplementary Table S2). All clean reads from male and female samples were combined into an assembly that generated 79,040 unigenes with a mean length of 773 bp and an N50 length of 1,711 bp. Based on size distribution analysis, 14,396 (18.21%) of the unigenes were longer than 1000 bp (Table 1).

**TABLE 1:** Summary of the transcriptome assembly of *P. saucia* antennae.

Length range (bp)	Transcript number (percentage)	Unigene number (percentage)
200−300	43,001 (28.68%)	34,135 (43.19%)
300−500	30,086 (20.06%)	19,167 (24.25%)
500−1000	25,729 (17.16%)	11,342 (14.35%)
1000−2000	22,358 (14.91%)	7,093 (8.97%)
2000+	28,784 (19.19%)	7,303 (9.24%)
Total number	149,958	79,040
Total length	188,244,110	61,110,725
N50 length	2,761	1,711
Mean length	1255.31	773.16

### GO Annotation and Classification

Unigenes were aligned using BLASTx to protein databases, including GO, Swiss-Prot, COG, KEGG, Pfam, and NR databases. A total of 20,004 (25.31%) unigenes were successfully annotated. Of the 79,040 unigenes, 18,639 (23.58%) had hits in the NR database with an *E*-value cut-off of 1e-5. Among the annotated unigenes, 13,533 (72.61%) had best matches to lepidopteran sequences, primarily *B. mori* (42.65%), *Danaus plexippus* (25.15%), and *H. armigera* (2.24%) (Supplementary Figure S1).

**FIGURE 1:**
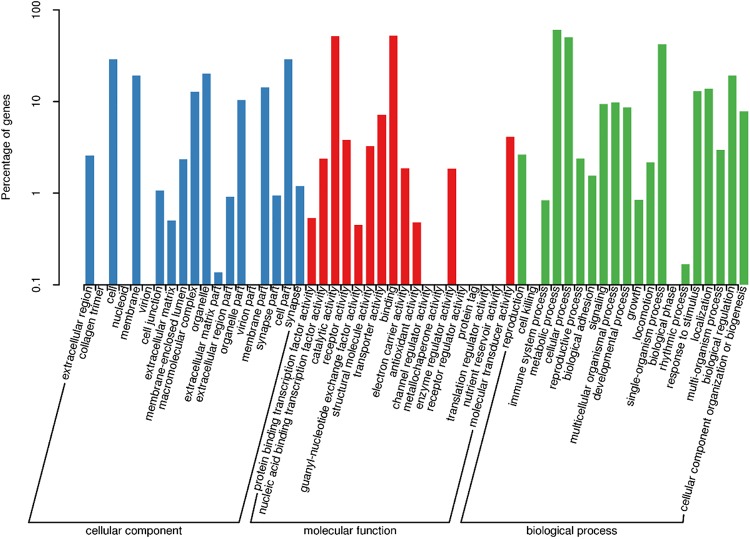
Functional annotation of *Peridroma saucia* antennae transcripts based on gene ontology (GO) categorization. The *Y*-axis shows the percentage of annotated GO terms in three categories: biological process, cellular component, and molecular function. The *X*-axis shows three areas of annotation, and in each area the sequences are further divided into subgroups.

Gene functional annotation was performed using Blast2GO to classify the sequences into functional groups according to GO category. Among the 79,040 unigenes, 24,820 (31.40%) identified sequences were allocated to at least one GO term. A total of 13,870 were assigned to a cellular component (17.54%), 12,488 to a molecular function (15.79%), and 23,880 to a biological process (30.21%). The most abundant and enriched GO term in the cellular component category were “cell” (2765 unigenes) and “cell part” (2765 unigenes). In the molecular function terms, “binding” (5004 unigenes) were the most represented. In the biological process terms, “metabolic process” (5833 unigenes) was shown to be the most abundant (Figure 1).

### Identification and Phylogenetic Analysis of Candidate ORs

Based on the sequence similarity to insect ORs, we identified 63 candidate OR genes in *P. saucia* antennae. Fifty of these *PsauOR* genes were putative full-length cDNAs encoding more than 379 amino acids and predicted to have 3−7 transmembrane domains (TMDs), which are characteristics of most insect ORs. The candidate PsauORs share between 49%–88% amino acid identity with published lepidopteran ORs in NCBI database, except for PsauORco, which shared 99% amino acid identity with *Mythimna separata* ORco. Details for the 63 ORs, including gene names, lengths, and BLASTx algorithm-based best hits are listed in Supplementary Table S3. All of these genes were submitted to the NCBI database, with accession numbers MN602154−MN602197, MN602199−MN602213, and MN602215−MN602218 (Supplementary Table S6).

**FIGURE 2:**
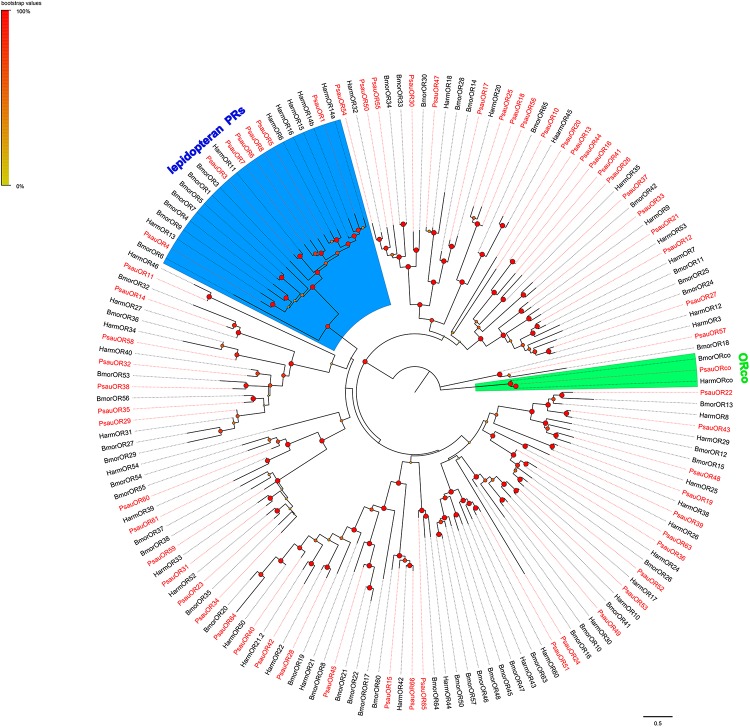
Maximum-likelihood tree of ORs from *P. saucia* and other Lepidoptera. The tree was rooted by the conservative ORco gene orthologs. Branches of the ORco clade are highlighted with green; branches containing “lepidopteran PRs” are highlighted with blue. Candidate PsauORs are colored with red letters. Node support was estimated with 1000 bootstrap replicates, and bootstrap values were displayed with circles at the branch nodes based on the scale indicated at the top left. The scale bar at the lower right indicates the branch length in proportion to amino acid substitutions per site. Psau, *P*. *saucia*; Harm, *H*. *armigera*; Bmor, *B*. *mori*.

A phylogenetic tree was constructed based on the alignment of candidate ORs from *P. saucia* (this study), *B. mori* (Wanner et al., 2007), and *H. armigera* (Zhang et al., 2015). As expected, ORcos of the three species were highly conserved and clustered in one branch. Seven ORs in *P. saucia*, including PsauOR1, PsauOR3, PsauOR4, PsauOR5, PsauOR6, PsauOR7, and PsauOR8, belonged to the lepidopteran pheromone receptors (PRs) clade (Figure 2).

### Identification and Phylogenetic Analysis of the Candidate GRs

We identified 10 putative GRs based on the bioinformatic analysis of the antennal transcriptome of *P. saucia* (GenBank accession numbers MN602219−MN602228, Supplementary Table S6). Among all identified PsauGRs, a complete ORF was identified in PsauGR1/2/4/5/6/7/8/10, while PsauGR3 and PsauGR9 were annotated as partial sequences. Like other typical insect GRs, *P. saucia* genes contain 6–8 TMDs, and their best hit GRs were from *H. armigera* and *Athetis dissimilis* (79%–81% identities) (Supplementary Table S4).

**FIGURE 3:**
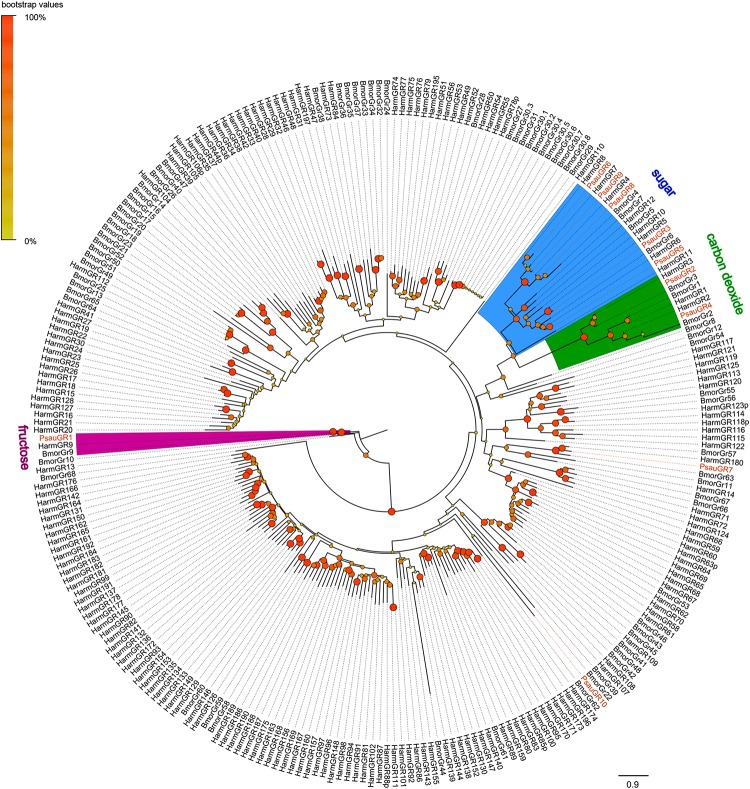
Maximum-likelihood tree of GRs from *P. saucia* and other Lepidoptera. The tree was rooted by the conservative BmorGR9 (fructose receptor) gene orthologs. Branches of the putative CO_2_ receptors are highlighted with green; branches of putative fructose receptors are highlighted with purple; branches containing “sugar-taste receptors” are highlighted with blue; and branches containing “bitted-taste receptors” are not highlighted. Node support was estimated with 1000 bootstrap replicates, and bootstrap values were displayed with circles at the branch nodes based on the scale indicated at the top left. Candidate PsauGRs are colored with red letters. The scale bar at the lower right indicates the branch length in proportion to amino acid substitutions per site. Psau, *P*. *saucia*; Harm, *H*. *armigera*; Bmor, *B*. *mori*.

A phylogenetic tree was constructed with GR sequences from *P. saucia*, *H. armigera*, and *B. mori*. PsauGR2 and PsauGR4, which grouped with BmorGR1/2/3 and HarmGR1/2/3, were putative candidate CO_2_ receptors. Five PsauGRs (PsauGR3/5/6/8/9) clustered with the BmorGR4/5/6/7/8 lineage, which detect sugar in *B. mori* (Wanner et al., 2007). In addition, PsauGR1 clustered with BmorGR9 and HarmGR9, which are fructose specific (Sato et al., 2011; Jiang et al., 2015). Only two GRs, PsauGR7, and PsauGR10, clustered in the clades containing putative bitter-compound receptors (Figure 3).

### Identification and Phylogenetic Analysis of the Candidate IRs/iGluRs

**FIGURE 4:**
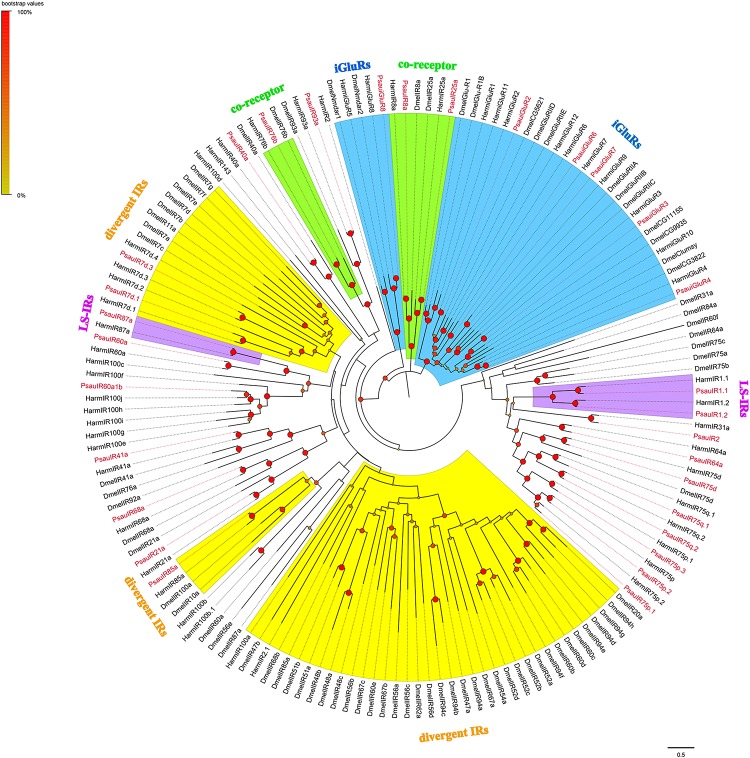
Maximum-likelihood tree of candidate IRs/iGluRs from *P. saucia*, *B. mori*, and *D. melanogaster*. The tree was rooted by the conservative iGluRs gene orthologs. Branches of IR co-receptors are highlighted with green; branches of the putative ionotropic glutamate receptors (iGluRs) are highlighted with blue; branches of the putative “divergent IRs” are highlighted with yellow; branches of the putative “Lepidoptera-specific IRs (LS-IRs)” are highlighted with purple; branches of the putative “antennal IRs” are not highlighted. Node support was estimated with 1000 bootstrap replicates, and bootstrap values were displayed with circles at the branch nodes based on the scale indicated at the top left. Candidate PsauIRs/iGluRs are colored with red letters. The scale bar at the lower right indicates the branch length in proportion to amino acid substitutions per site. Psau, *P*. *saucia*; Dmel, *D*. *melanogaster*; Bmor, *B*. *mori*.

A total of 24 candidate PsauIRs and 6 PsauiGluRs were identified from the antennal transcriptome (GenBank accession numbers MN602229−MN602258, Supplementary Table S6). Among these candidate genes, full-length ORFs with 3–6 TMDs were identified for 24 IRs/iGluRs, whereas the other 6 IRs/iGluRs were partial sequences (Supplementary Table S5). According to the maximum-likelihood tree of IRs from *P. saucia*, *H. armigera*, and *D. melanogaster*, the putative co-receptors of *P. saucia* PsauIR8a, PsauIR25a, and Psau76b clustered within the highly conserved co-receptor lineages of DmelIR8a, DmelIR25a, and Dmel76b, respectively. Six iGluRs identified from *P. saucia* clustered in the large sub-families of the iGluRs clade. We also identified three PsauIRs (PsauIR1.1/1.2/87a) belonging to the “Lepidoptera-specific” subfamilies IR1 and IR87a. Most PsauIRs belong to presumed “antennal IR” orthologs based on tissue expression patterns in insects, except for PsauIR7d.1, PsauIR7d.3, and PsauIR85a, which were in the “divergent IRs” clade (Figure 4).

### RT-qPCR Verification of Candidate ORs, GRs, and IRs/iGluRs

**FIGURE 5:**
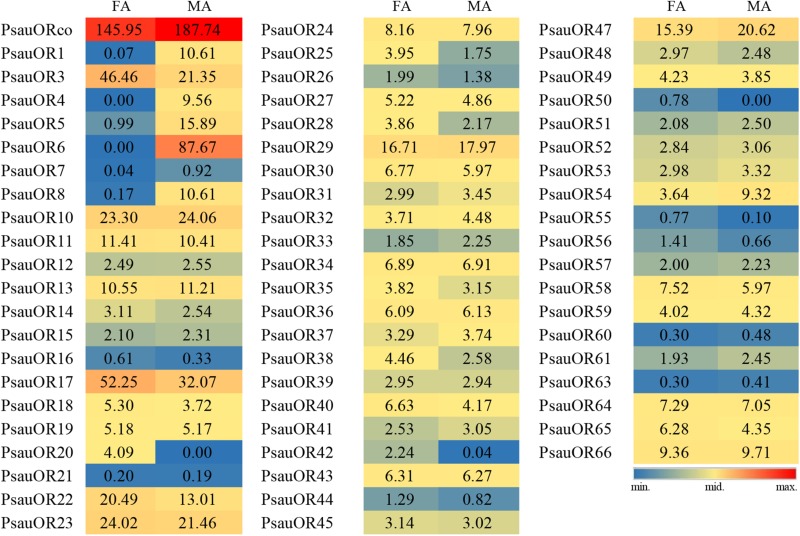
Heat-plot of FPKM values for PsauORs in female antennae (FA) and male antennae (MA). In each box, the FPKM value of each PsauOR gene is indicated. Color scales were established for PsauORs using the conditional formatting option in Excel (red: max. value, yellow: mid. value, and blue: min. value).

**FIGURE 6:**
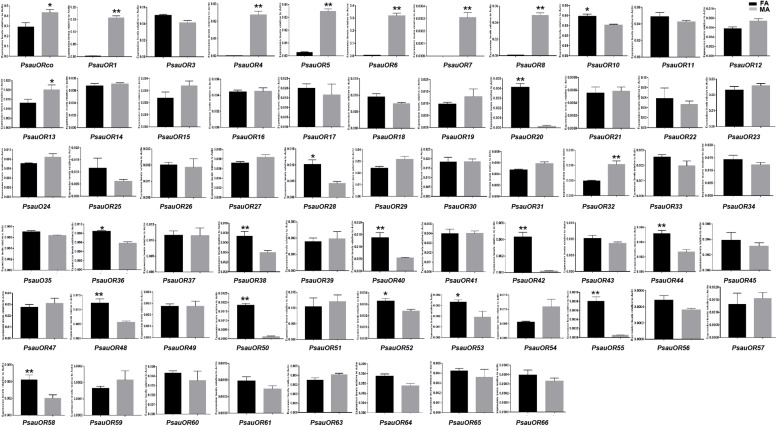
Expression patterns of candidate *ORs* in *P. saucia*. RT-qPCR analysis was conducted for candidate *OR* genes in female antennae (FA) and male antennae (MA). (Student’s *t*-test, error bars indicate standard errors of the means; **P < 0.01; *P < 0.05; *n* = 3).

To validate and analyze the expression differences of candidate chemosensory receptor genes between male antennae (MA) and female antennae (FA), all candidate chemosensory receptor genes encoding ORs, GRs, and IRs/iGluRs were subjected to RT-qPCR. Expression patterns of the 103 chemoreceptors were basically consistent with the FPKM values in female and male antennae. According to the RT-qPCR results, the expression levels of 23 of the 63 candidate OR genes significantly differed between male and female antennae (*P* < 0.05). Among these 23 genes, expression levels of *PsauORco*, *PsauOR13*, and *PsauOR32* were higher in male than in female antennae; 6 OR genes (*PsauOR1*/*4*/*5*/*6*/*7*/*8*) were predominantly expressed in male antennae; and expression levels of 14 OR genes (*PsauOR10*/*20*/*28*/*36*/*38/40*/*42*/*44*/*48*/*50*/*52*/*53*/*55*/*58*) were higher in female than male antennae. Expression of the other 40 *PsauORs* did not significantly differ between two sexes (*P* < 0.05) (Figures 5, 6).

**FIGURE 7:**
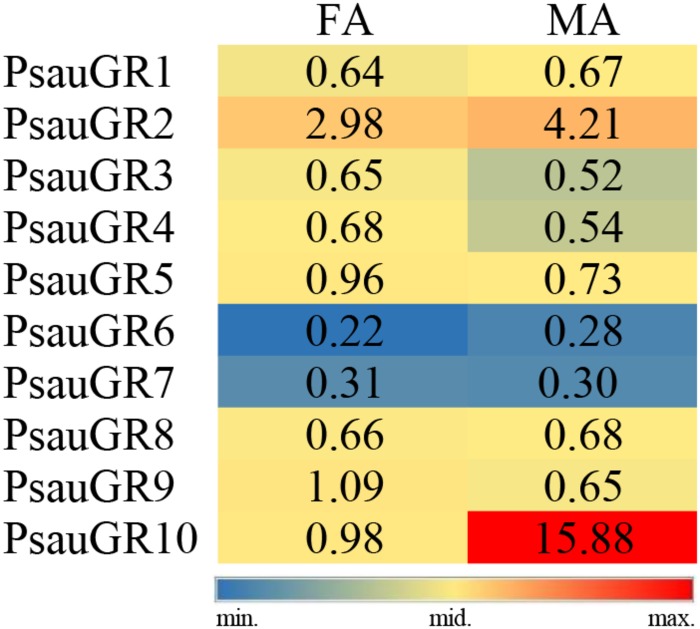
Heat-plot of FPKM values for PsauGRs in female antennae (FA) and male antennae (MA). In each box, the FPKM value of each PsauGR gene is indicated. Color scales were established for PsauGRs using the conditional formatting option in Excel (red: max. value, yellow: mid. value, and blue: min. value).

**FIGURE 8:**
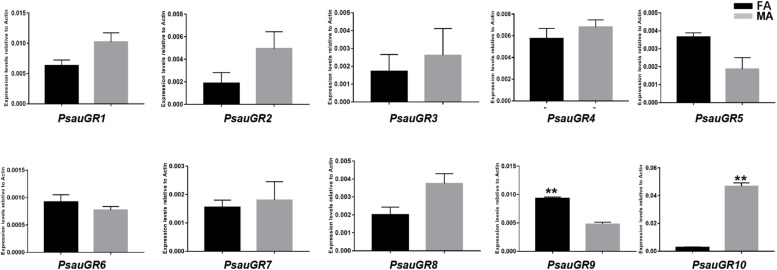
Expression patterns of candidate *GRs* in *P. saucia*. RT-qPCR analysis was conducted for candidate *GR* genes in female antennae (FA) and male antennae (MA). (Student’s *t*-test, error bars indicate standard errors of the means; **P < 0.01; *n* = 3).

Among GR genes, the expression of *PsauGR9* was significantly higher in female antennae, whereas the expression of *PsauGR10* was significantly higher in male antennae (*P* < 0.05) (Figures 7, 8).

**FIGURE 9:**
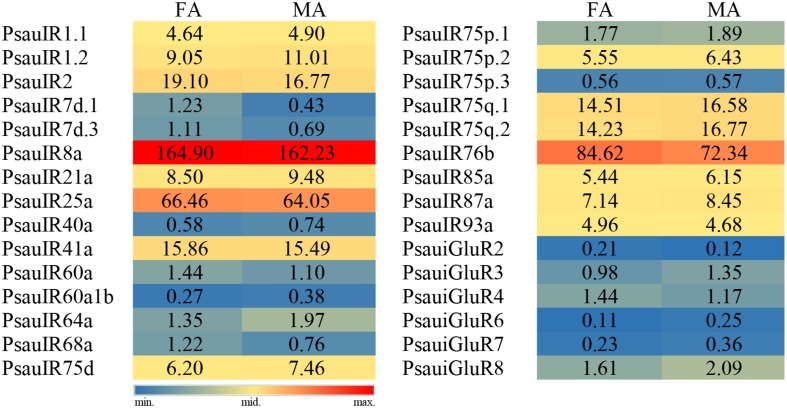
Heat-plot of FPKM values for PsauIRs/iGluRs in female antennae (FA) and male antennae (MA). In each box, the FPKM value of each PsauIR/iGluR gene is indicated. Color scales were established for PsauIRs/iGluRs using the conditional formatting option in Excel (red: max. value, yellow: mid. value, and blue: min. value).

**FIGURE 10:**
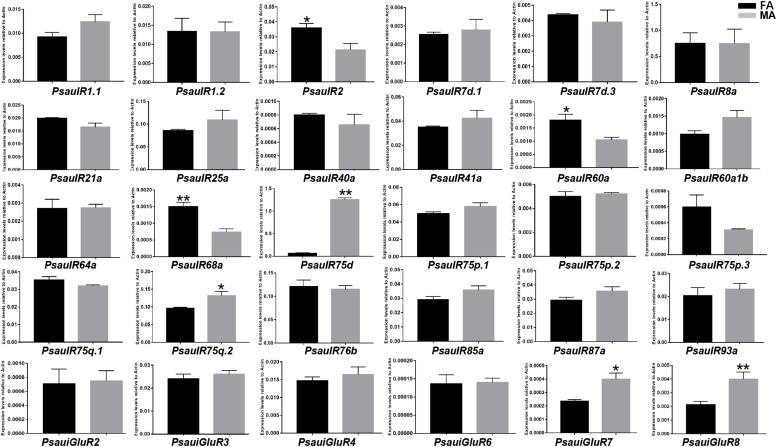
Expression patterns of candidate *IRs*/*iGluRs* in *P. saucia*. RT-qPCR analysis was conducted for candidate *IR* genes in female antennae (FA) and male antennae (MA). (Student’s *t*-test, error bars indicate standard errors of the means; **P < 0.01; *P < 0.05; *n* = 3).

The expression of most *PsauIR*s/*iGluRs* did not significantly differ between male and female antennae. However, the expression of 3 IR genes (*PsauIR2*/*60a*/*68a*) was higher in female antennae, and that of 4 IR/iGluR genes (*PsauIR75d*/*75q.2* and *PsauiGluR7*/*8*) was higher in male antennae (Figures 9, 10) (*P* < 0.05).

## Discussion

In this study, we reported on the sequencing, assembly, and annotation of the antennal transcriptome of the polyphagous crop pest *P. saucia*. We identified 63 ORs, 10 GRs, 24 IRs, and 6 iGluRs. The number of identified chemoreceptor genes is comparable to that reported for the lepidopteran antennal transcriptomes of *Spodoptera littoralis* (60 ORs, 17 GRs, and 17 IRs) and *Galleria mellonella* (46 ORs and 25 IRs) (Walker et al., 2019; Zhao et al., 2019). Of the 103 chemoreceptors reported in the current study, 79.61% (*n* = 82) have been predicted as complete ORF encoding cDNAs, which provides high confidence in the quality of the transcriptome sequencing.

As the centerpiece of peripheral olfactory reception, ORs are the most important and determine the sensitivity and specificity of odorant reception (Leal, 2013). Genomic studies of the odorant receptors in several moth/butterfly species have reported 71 ORs in *B. mori* (Wanner et al., 2007), 73 in *Manduca sexta* (Koenig et al., 2015), 84 in *H. armigera* (Pearce et al., 2017), 74 in *Heliconius melpomene* (Dasmahapatra et al., 2012), and 64 in *D. plexippus* (Zhan et al., 2011). A total of 63 *PsauOR*s were annotated in our research, indicating that we have identified nearly the full repertoire of ORs in this species. Previous research has suggested that ORco may be the most highly expressed ORs in insect antennae (Jones et al., 2005; Sun et al., 2019), and a high expression of ORco was also documented in the current study of *P. saucia*. According to the FPKM values and the RT-qPCR results, *PsauORco* had the highest expression levels among all of the annotated ORs in *P. saucia* antennae. Moreover, *PsauORco* appeared to be expressed at a higher level in male antennae than in female antennae, which was not in accordance with some previous studies reporting similar expression levels of ORco between males and females (Krieger et al., 2003; Zhang et al., 2010). The skewed expression of ORco in male antennae may reflect a higher degree of sexual dimorphism in the distribution of trichoid sensilla between male and female antennae of *P*. *saucia*. Seven PsauORs (PsauOR1/3/4/5/6/7/8) clustered in the moth PR-subfamily (Wanner et al., 2007; Zhang et al., 2015), suggesting that these ORs are putative pheromone receptors specifically functioning in sexual communication. Besides, expression levels of *PsauOR1*, *PsauOR4*, *PsauOR5*, *PsauOR6*, *PsauOR7*, and *PsauOR8* are much higher in male than in female antennae, suggesting these PsauORs respond to components of female sex pheromones (Inomata et al., 2002; Choi et al., 2009). Other PsauORs, which had relatively low similarities with PRs, may be associated with detection of host plant odors. Those PsauORs with higher expression in female than in male antennae are likely to function in the detection of oviposition-related plant odors. Those PsauORs expressed at similar levels in male and female antennae are likely to function in food source odors perception.

Members of the GR family, which are usually abundant in the gustatory organs of insects, function in perceiving CO_2_, sugar, bitter substances, and other nutrients (Clyne et al., 2000). We identified 10 GRs in the *P. saucia* antennal transcriptome. This number is far lower than reported for other lepidopterans. Analyses of the *H. armigera* genome, for example, revealed a GR family of 197 genes (Xu et al., 2016). The number of GR family genes in another Noctuidae species, *S. frugiperda*, was 230 (Gouin et al., 2017). The low number of GRs identified in the current study might be explained by the fact that GR genes are mainly expressed in gustatory organs including tarsi, mouthparts, and ovipositors, rather than in antennae. CO_2_ is important in the foraging and oviposition of phytophagous insects (Guerenstein and Hildebrand, 2008). Specialized receptor cells that detect CO_2_ are located in the labial palps in lepidopteran adults (Bogner et al., 1986; Ning et al., 2016). In the current study of *P. saucia*, the expression levels of two identified CO_2_ GRs (*PsauGR2/4*) were similar in male and female antennae. Further work is required to define the molecular mechanisms and functional role of CO_2_ detection in *P. saucia*.

Five *P. saucia* GRs (PsauGR3/5/6/8/9) were determined in the clade of putative sugar receptors. Genome analyses and transcriptome sequencing have been used to characterize the repertoires of this highly conserved GR sub-family in a number of lepidopteran species. For example, five receptors for sugar-compounds were reported in *S*. *littoralis* and *B*. *mori* (Wanner and Robertson, 2008; Walker et al., 2019), and seven were reported in *H. armigera* (Xu et al., 2017). Although excellent progress has been made in understanding the role of the insect GR family in taste perception, most research has involved the model organism *D. melanogaster*. However, members from the fructose sub-family have been well-studied in moth species, such as HarmGR9 in *H. armigera* (Jiang et al., 2015) and BmorGR9 in *B. mori* (Sato et al., 2011). They have been shown to be responsive to fructose in heterologous experiment. We identified a GR gene (PsauGR1) that clusters with other fructose-receptors in this clade. Expression of *PsauGR1* was detected in both male and female antennae, and the amino acid identities of PsauGR1 with BmorGR9 and HarmGR9 were 64 and 90%, respectively, suggesting that PsauGR1 might be responsible for antennal fructose detection.

The sub-family of “bitter receptors” mainly participates in the perception of the large variety of secondary plant chemicals that caterpillars and moths encounter (Wanner and Robertson, 2008). Recent transcriptomic and genomic data from moth species have suggested that the expansion in the bitter-taste GR family may be functionally related to the behavior of polyphagous moths (Xu et al., 2016; Gouin et al., 2017). Because *P. saucia* is highly polyphagous, identification and characterization of putative bitter-taste GRs in other taste organs of *P*. *saucia* are still necessary.

Another type of chemosensory receptor, IR, is a conserved family that functions in the detection of acids, amines, aldehydes, sex pheromones, and also in gustation, thermosensation, and hygrosensation (Benton et al., 2009). Based on antennal transcriptome sequencing, we identified 24 IRs and 6 iGluRs in *P. saucia*. The putative IR co-receptors (*PsauIR8a*, *PsauIR25a*, and *PsauIR76b*) displayed higher expression than other IRs, which was consistent with other studies (Du et al., 2018; Walker et al., 2019; Zhao et al., 2019). According to the phylogenetic tree, six putative PsauiGluRs clustered with *D. melanogaster* and *H. armigera* iGluRs. In addition, IR members of the “Lepidoptera-specific” subfamilies (IR1 and IR87a) also occur in *P. saucia*. Although “divergent IRs” were reported as the largest sub-group in *D. melanogaster* (Croset et al., 2010), we only found three such ionotropic receptors in *P. saucia* antennae. In contrast, we found 15 PsauIRs in the “antennal IRs” subgroup. This difference can probably be explained by the fact that that we annotated IRs from antennae but not from other olfactory or gustatory tissues. Based on RT-qPCR results, *PsauIR2*, *PsauIR60a*, *PsauIR68a*, *PsauIR75d*, *PsauIR75q.2*, *PsauiGluR7*, and *PsauiGluR8* were expressed more in male than female antennae or vice versa. We speculate that these receptors may be involved in the perception of sex-related pheromones or other olfactory/contact compounds.

In summary, we used Illumina sequencing to analyze the transcriptomes of antennae of the variegated cutworm *P. saucia*. We annotated 63 ORs, 10 GRs, 24 IRs, and 6 iGluRs. We then used RT-qPCR to compare the expression of these genes in male and female antennae. The results provide a foundation for future research on the chemosensory system of *P. saucia* at the molecular level, and should also facilitate the study of molecular mechanisms and evolution of chemosensation in other Noctuidae species.

## Data Availability Statement

The datasets generated for this study can be found in the MN602154–MN6022197, MN602199–MN602213, MN602154–MN602218, MN602219–MN602228, and MN602229– MN602258.

## Author Contributions

S-LW and J-FD conceived and designed the study. Y-LS, J-FD, and NG collected the biological material, performed the transcriptome data analysis, and constructed the phylogenetic trees. Y-LS and S-LW performed the molecular work. Y-LS wrote the manuscript. All authors read and approved the final version of the manuscript.

## Conflict of Interest

The authors declare that the research was conducted in the absence of any commercial or financial relationships that could be construed as a potential conflict of interest.

## Footnotes

1 http://www.ncbi.nlm.nih.gov/

2 http://www.ebi.ac.uk/uniprot

3 http://kobas.cbi.pku.edu.cn/

4 https://www.ncbi.nlm.nih.gov/orffinder/

5 https://web.expasy.org/translate/

6 http://www.cbs.dtu.dk/services/TMHMM/

7 http://tree.bio.ed.ac.uk/software/figtree

## Supplementary Material

FIGURE S1 Nr Homologous species distribution.

TABLE S1 Primers for real-time quantitative-PCR of candidate ORs, GRs, and IR/iGluRs in *P. saucia*.

TABLE S2 Evaluation of sequencing data of *P. saucia* samples.

TABLE S3 Details for candidate odorant receptors in *P. saucia* antennae.

TABLE S4 Details for candidate gustatory receptors in *P. saucia* antennae.

TABLE S5 Details for candidate IRs/iGluRs in *P. saucia* antennae.

TABLE S6 Names, GenBank accession numbers and sequences of PsauORs/GRs/IRs/iGluRs.

## References

[B1] Abuin L. Bargeton B. Ulbrich M. H. Isacoff E. Y. Kellenberger S. Benton R. (2011). Functional architecture of olfactory ionotropic glutamate receptors. *Neuron* 69 44–60. 10.1016/j.neuron.2010.11.042 21220098 PMC3050028

[B2] Altschul S. F. Madden T. L. Schäffer A. A. Zhang J. Zhang Z. Miller W. (1997). Gapped BLAST and PSI-BLAST: a new generation of protein database search programs. *Nucleic Acids Res.* 25 3389–3402. 10.1093/nar/25.17.3389 9254694 PMC146917

[B3] Ashburner M. Ball C. A. Blake J. A. Botstein D. Butler H. Cherry J. M. (2000). Gene ontology: tool for the unification of biology. *Nat. Genet.* 25 25–29. 10.1038/75556 10802651 PMC3037419

[B4] Benton R. Vannice K. S. Gomez-Diaz C. Vosshall L. B. (2009). Variant ionotropic glutamate receptors as chemosensory receptors in *Drosophila*. *Cell* 136 149–162. 10.1016/j.cell.2008.12.001 19135896 PMC2709536

[B5] Bogner F. Boppré M. Ernst K. D. Boeckh J. (1986). CO2 sensitive receptors on labial palps of *Rhodogastria* moths (Lepidoptera: Arctiidae): physiology, fine structure and central projection. *J. Comp. Physiol. A* 158 741–749. 10.1007/bf01324818 3090241

[B6] Capinera J. L. Pelissier D. Menout G. S. Epsky N. D. (1988). Control of black cutworm, *Agrotis ipsilon* (Lepidoptera: Noctuidae), with entomogenous nematodes (Nematoda: Steinernematidae, Heterorhabditidae). *J. Invertebr. Pathol.* 52 427–435. 10.1016/0022-2011(88)90055-9

[B7] Chen C. Buhl E. Xu M. Croset V. Rees J. S. Lilley K. S. (2015). *Drosophila* ionotropic receptor 25a mediates circadian clock resetting by temperature. *Nature* 527 516–520. 10.1038/nature16148 26580016

[B8] Choi K. S. Cho J. R. Song J. H. Kim D. S. Boo K. S. (2009). Sex pheromone composition of the variegated cutworm, *Peridroma saucia* (Lepidoptera: Noctuidae), in Korea. *J. Asia Pac. Entomol.* 12 71–77. 10.1016/j.aspen.2009.01.004

[B9] Clyne P. J. Warr C. G. Carlson J. R. (2000). Candidate taste receptors in *Drosophila*. *Science* 287 1830–1834. 10.1126/science.287.5459.1830 10710312

[B10] Clyne P. J. Warr C. G. Freeman M. R. Lessing D. Kim J. Carlson J. R. (1999). A novel family of divergent seven-transmembrane proteins: candidate odorant receptors in *Drosophila*. *Neuron* 22 327–338. 10.1016/S0896-6273(00)81093-4 10069338

[B11] Croset V. Rytz R. Cummins S. F. Budd A. Brawand D. Kaessmann H. (2010). Ancient protostome origin of chemosensory ionotropic glutamate receptors and the evolution of insect taste and olfaction. *PLoS Genet.* 6:e1001064. 10.1371/journal.pgen.1001064 20808886 PMC2924276

[B12] Dasmahapatra K. K. Walters J. R. Briscoe A. D. Davey J. W. Whibley A. Nadeau N. J. (2012). Butterfly genome reveals promiscuous exchange of mimicry adaptations among species. *Nature* 487 94–98. 10.1038/nature11041 22722851 PMC3398145

[B13] Deng Y. Y. Li J. Q. Wu S. F. Zhu Y. P. He F. C. (2006). Integrated nr database in protein annotation system and its localization. *Comput. Eng.* 32 71–72. 10.1109/INFOCOM.2006.241

[B14] Du L. X. Liu Y. Zhang J. Gao X. W. Wang B. Wang G. R. (2018). Identification and characterization of chemosensory genes in the antennal transcriptome of *Spodoptera exigua*. *Comp. Biochem. Phys. D Genomics Proteomics* 27 54–65. 10.1016/j.cbd.2018.05.001 29787920

[B15] Engsontia P. Sanderson A. P. Cobb M. Walden K. K. O. Robertson H. M. Brown S. (2008). The red flour beetle’s large nose: an expanded odorant receptor gene family in *Tribolium castaneum*. *Insect. Biochem. Mol. Biol.* 38 387–397. 10.1016/j.ibmb.2007.10.005 18342245

[B16] Enjin A. Zaharieva E. E. Frank D. D. Mansourian S. Suh G. S. Gallio M. (2016). Humidity sensing in *Drosophila*. *Curr. Biol.* 26 1352–1358. 10.1016/j.cub.2016.03.049 27161501 PMC5305172

[B17] Fleischer J. Pregitzer P. Breer H. Krieger J. (2018). Access to the odor world: odorant receptors and their role for signal transduction in insects. *Cell. Mol. Life Sci.* 75 485–508. 10.1007/s00018-017-2627-5 28828501 PMC11105692

[B18] Frank D. D. Enjin A. Jouandet G. C. Zaharieva E. E. Para A. Stensmyr M. C. (2017). Early integration of temperature and humidity stimuli in the *Drosophila* brain. *Curr. Biol.* 27 2381–2388. 10.1016/j.cub.2017.06.077 28736172 PMC5600489

[B19] Gao Q. Chess A. (1999). Identification of candidate *Drosophila* olfactory receptors from genomic DNA sequence. *Genomics* 60 31–39. 10.1006/geno.1999.5894 10458908

[B20] Gouin A. Bretaudeau A. Nam K. Gimenez S. Aury J. M. Duvic B. (2017). Two genomes of highly polyphagous lepidopteran pests (*Spodoptera frugiperda*, Noctuidae) with different host-plant ranges. *Sci. Rep.* 7:11816. 10.1038/s41598-017-10461-4 28947760 PMC5613006

[B21] Guerenstein P. G. Hildebrand J. G. (2008). Roles and effects of environmental carbon dioxide in insect life. *Annu. Rev. Entomol.* 53 161–178. 10.1146/annurev.ento.53.103106.093402 17803457

[B22] Guo M. Sui H. Han H. L. (2010). Two new record species of Noctuinae (Lepidoptera, Noctuidae) from Northeast China. *J. Northeast For. Univ.* 38 129–130. 10.11646/zootaxa.4609.3.11 31717101

[B23] Hansson B. S. Stensmyr M. C. (2011). Evolution of insect olfaction. *Neuron* 72 698–711. 10.1016/j.neuron.2011.11.003 22153368

[B24] Inomata S. I. Tsuchiya S. Ikeda K. Saito O. Ando T. (2002). Identification of the sex pheromone components secreted by female moths of *Peridroma saucia* (Noctuidae: Noctuinae). *Biosci. Biotechnol. Biochem.* 66 2461–2464. 10.1271/bbb.66.2461 12506988

[B25] Jiang X. J. Ning C. Guo H. Jia Y. Y. Huang L. Q. Qu M. J. (2015). A gustatory receptor tuned to D-fructose in antennal sensilla chaetica of *Helicoverpa armigera*. *Insect Biochem. Mol. Biol.* 60 39–46. 10.1016/j.ibmb.2015.03.002 25784630

[B26] Jones D. T. Taylor W. R. Thornton J. M. (1992). The rapid generation of mutation data matrices from protein sequences. *Comput. Appl. Biosci.* 8 275–282. 10.1093/bioinformatics/8.3.275 1633570

[B27] Jones W. D. Nguyen T. A. Kloss B. Lee K. J. Vosshall L. B. (2005). Functional conservation of an insect odorant receptor gene across 250 million years of evolution. *Curr. Biol.* 15 R119–R121. 10.1016/j.cub.2005.02.007 15723778

[B28] Kanehisa M. (2004). The KEGG resource for deciphering the genome. *Nucleic Acids Res.* 32 277–280. 10.1093/nar/gkh063 14681412 PMC308797

[B29] Knecht Z. A. Silbering A. F. Ni L. Klein M. Budelli G. Bell R. (2016). Distinct combinations of variant ionotropic glutamate receptors mediate thermosensation and hygrosensation in *Drosophila*. *eLife* 5:e17879. 10.7554/eLife.17879 27656904 PMC5052030

[B30] Koenig C. Hirsh A. Bucks S. Klinner C. Vogel H. Shukla A. (2015). A reference gene set for chemosensory receptor genes of *Manduca sexta*. *Insect Biochem. Mol. Biol.* 66 51–63. 10.1016/j.ibmb.2015.09.007 26365739

[B31] Krieger J. Klink O. Mohl C. Raming K. Breer H. (2003). A candidate olfactory receptor subtype highly conserved across different insect orders. *J. Comp. Physiol. A Neuroethol. Sens. Neural. Behav. Physiol.* 189 519–526. 10.1007/s00359-003-0427-x 12827420

[B32] Kuang C. C. (1985). Studies on the biology and control of the *Peridroma saucia*. *Chi. Bull. Entomol.* 2 16–19.

[B33] Kumar S. Stecher G. Tamura K. (2016). MEGA7: molecular evolutionary genetics analysis version 7.0 for bigger datasets. *Mol. Biol. Evol.* 33 1870–1874. 10.1093/molbev/msw054 27004904 PMC8210823

[B34] Kwon J. Y. Dahanukar A. Weiss L. A. Carlson J. R. (2007). The molecular basis of CO2 reception in *Drosophila*. *Proc. Natl. Acad. Sci. U.S.A.* 104 3574–3578. 10.1073/pnas.0700079104 17360684 PMC1805529

[B35] Leal W. S. (2013). Odorant reception in insects: roles of receptors, binding proteins, and degrading enzymes. *Annu. Rev. Entomol.* 58 373–391. 10.1146/annurev-ento-120811-153635 23020622

[B36] Li M. Tan J. C. Gu Z. R. Song B. D. Liu J. Teng K. (2007). Record of moths in Badagongshan National nature reserve. *J. Biosaf.* 16 290–298.

[B37] Liu N. Y. Xu W. Dong S. L. Zhu J. Y. Xu Y. X. Anderson A. (2018). Genome-wide analysis of ionotropic receptor gene repertoire in Lepidoptera with an emphasis on its functions of *Helicoverpa armigera*. *Insect Biochem. Mol. Biol.* 99 37–53. 10.1016/j.ibmb.2018.05.005 29800678

[B38] Ning C. Yang K. Xu M. Huang L. Q. Wang C. Z. (2016). Functional validation of the carbon dioxide receptor in labial palps of *Helicoverpa armigera* moths. *Insect Biochem. Mol. Biol.* 73 12–19. 10.1016/j.ibmb.2016.04.002 27060445

[B39] Pearce S. L. Clarke D. F. East P. D. Elfekih S. Gordon K. H. J. Jermiin L. S. (2017). Genomic innovations, transcriptional plasticity and gene loss underlying the evolution and divergence of two highly polyphagous and invasive *Helicoverpa* pest species. *BMC Biol.* 15:63. 10.1186/s12915-017-0402-6 28756777 PMC5535293

[B40] Rings R. W. Johnson B. A. Arnold F. J. (1976). Host range of the variegated cutworm on vegetables: a bibliography. *Bull. Entomol. Soc. Am.* 22 409–415. 10.1093/besa/22.4.409

[B41] Sato K. Pellegrino M. Nakagawa T. Vosshall L. B. Touhara K. (2008). Insect olfactory receptors are heteromeric ligand-gated ion channels. *Nature* 452 1002–1006. 10.1038/nature06850 18408712

[B42] Sato K. Tanaka K. Touhara K. (2011). Sugar-regulated cation channel formed by an insect gustatory receptor. *Proc. Natl. Acad. Sci. U.S.A.* 108 11680–11685. 10.1073/pnas.1019622108 21709218 PMC3136286

[B43] Schmittgen T. D. Livak K. J. (2008). Analyzing real-time PCR data by the comparative CT method. *Nat. Protoc.* 3 1101–1108. 10.1038/nprot.2008.73 18546601

[B44] Scott K. Brady R. Jr. Cravchik A. Morozov P. Rzhetsky A. Zuker C. (2001). A chemosensory gene family encoding candidate gustatory and olfactory receptors in *Drosophila*. *Cell* 104 661–673. 10.1016/s0092-8674(01)00263-x 11257221

[B45] Simonet D. E. Clemet S. L. Rubik W. L. Rings R. W. (1981). Temperature requirements for development and oviposition of *Peridroma saucia* (Lepidoptera: Noctuidae). *Can. Entomol.* 113 891–897. 10.4039/Ent113891-10

[B46] Struble D. L. Swailes G. E. Steck W. F. Underhill E. W. Chisholm M. D. (1976). A sex attractant for adult males of variegated cutworm, *Peridroma saucia*. *Environ. Entomol.* 5 988–990. 10.1093/ee/5.5.988

[B47] Sun Y. L. Dong J. F. Ning C. Ding P. P. Huang L. Q. Sun J. G. (2019). An odorant receptor mediates the attractiveness of *cis*-jasmone to *Campoletis chlorideae*, the endoparasitoid of *Helicoverpa armigera*. *Insect Mol. Biol.* 28 23–34. 10.1111/imb.12523 30058747

[B48] Thompson J. D. Higgins D. G. Gibson T. J. (1994). CLUSTALW: improving the sensitivity of progressive multiple sequence alignment through sequence weighting, position-specific gap penalties and weight matrix choice. *Nucleic Acids Res.* 22 4673–4680. 10.1093/nar/22.22.4673 7984417 PMC308517

[B49] Vosshall L. Amrein H. Morozov P. Rzhetsky A. Axel R. (1999). A spatial map of olfactory receptor expression in the *Drosophila* antenna. *Cell* 96 725–736. 10.1016/S0092-8674(00)80582-6 10089887

[B50] Walker W. B. III Roy A. Anderson P. Schlyter F. Hansson B. S. Larsson M. C. (2019). Transcriptome analysis of gene families involved in chemosensory function in *Spodoptera littoralis* (Lepidoptera: Noctuidae). *BMC Genomics* 20:428. 10.1186/s12864-019-5815-x 31138111 PMC6540431

[B51] Wanner K. W. Anderson A. R. Trowell S. C. Theilmann D. A. Robertson H. M. Newcomb R. D. (2007). Female-biased expression of odorant receptor genes in the adult antennae of the silkworm, *Bombyx mori*. *Insect Mol. Biol.* 16 107–119. 10.1111/j.1365-2583.2007.00708.x 17257213

[B52] Wanner K. W. Robertson H. M. (2008). The gustatory receptor family in the silkworm moth *Bombyx mori* is characterized by a large expansion of a single lineage of putative bitter receptors. *Insect Mol. Biol.* 17 621–629. 10.1111/j.1365-2583.2008.00836.x 19133074

[B53] Wicher D. Schäfer R. Bauernfeind R. Stensmyr M. C. Heller R. Heinemann S. H. (2008). *Drosophila* odorant receptors are both ligand-gated and cyclic-nucleotide-activated cation channels. *Nature* 452 1007–1011. 10.1038/nature06861 18408711

[B54] Willson H. R. Semel M. Tebcherany M. Prostak D. J. Hill A. S. (1981). Evaluation of sex attractant and blacklight traps for monitoring black cutworm and variegated cutworm. *J. Econ. Entomol.* 74 517–519. 10.1093/jee/74.5.517

[B55] Xie C. Mao X. Huang J. Ding Y. Wu J. Dong S. (2011). KOBAS 2.0: a web server for annotation and identification of enriched pathways and diseases. *Nucleic Acids Res.* 39 316–322. 10.1093/nar/gkr483 21715386 PMC3125809

[B56] Xu W. Liu N. Liao Y. Anderson A. (2017). Molecular characterization of sugar taste receptors in the cotton bollworm *Helicoverpa armigera*. *Genome* 60 1037–1044. 10.1139/gen-2017-0086 28825966

[B57] Xu W. Papanicolaou A. Zhang H. J. Anderson A. (2016). Expansion of a bitter taste receptor family in a polyphagous insect herbivore. *Sci. Rep.* 6:23666. 10.1038/srep23666 27032373 PMC4817054

[B58] Xuan S. B. Zhang Q. Wang H. Shi B. M. Yue J. Y. Wang J. P. (2012). Report II on Noctuidae (Lepidoptera) in the headstreams of Fenhe River, Shanxi Province. *J. Shanxi Agric. Sci.* 39 1092–1095.

[B59] Zhan S. Merlin C. Boore J. L. Reppert S. M. (2011). The monarch butterfly genome yields insights into long-distance migration. *Cell* 147 1171–1185. 10.1016/j.cell.2011.09.052 22118469 PMC3225893

[B60] Zhang D. D. Zhu K. Y. Wang C. Z. (2010). Sequencing and characterization of six cDNAs putatively encoding three pairs of pheromone receptors in two sibling species, *Helicoverpa armigera* and *Helicoverpa assulta*. *J. Insect Physiol.* 56 586–593. 10.1016/j.jinsphys.2009.12.002 19962987

[B61] Zhang J. Wang B. Dong S. L. Cao D. Dong J. F. Walker W. B. (2015). Antennal transcriptome analysis and comparison of chemosensory gene families in two closely related noctuidae moths, *Helicoverpa armigera* and *H. assulta*. *PLoS One* 10:e0117054. 10.1371/journal.pone.0117054 25659090 PMC4319919

[B62] Zhao H. X. Xiao W. Y. Ji C. H. Ren Q. Xia X. S. Zhang X. F. (2019). Candidate chemosensory genes identified from the greater wax moth, *Galleria mellonella*, through a transcriptomic analysis. *Sci. Rep.* 9:10032. 10.1038/s41598-019-46532-x 31296896 PMC6624281

[B63] Zhou X. Rokas A. Berger S. L. Liebig J. Ray A. Zwiebel L. J. (2015). Chemoreceptor evolution in Hymenoptera and its implications for the evolution of eusociality. *Genome Biol. Evol.* 7 2407–2416. 10.1093/gbe/evv149 26272716 PMC4558866

