# Supplementary figures and images for: Identification of Candidate Chemosensory Receptors in the Antennae of the Variegated Cutworm, Peridroma saucia Hübner, Based on a Transcriptome Analysis
Source: Front Physiol. 2020 Jan 31;11:39. doi: 10.3389/fphys.2020.00039 (PMC7005060; doi:10.3389/fphys.2020.00039)

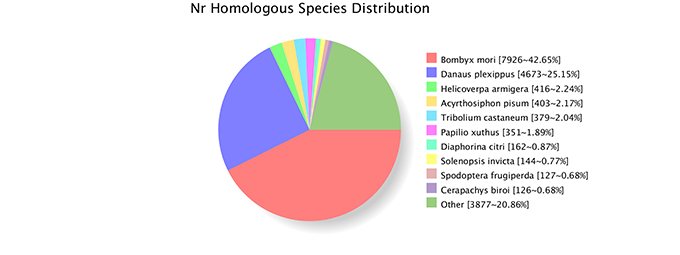

Supplement: FIGURE S1 — Nr Homologous species distribution. [file Image_1.TIF]
