# Supplementary material for: Identification of Candidate Chemosensory Receptors in the Antennae of the Variegated Cutworm, Peridroma saucia Hübner, Based on a Transcriptome Analysis
Source: Front Physiol. 2020 Jan 31;11:39. doi: 10.3389/fphys.2020.00039 (PMC7005060; doi:10.3389/fphys.2020.00039)
Supplement: TABLE S1 — Primers for real-time quantitative-PCR of candidate ORs, GRs, and IR/iGluRs in P. saucia. [file Table_1.docx]

**Table S1**. Primers for real-time quantitative-PCR of candidate *OR*s, *GRs*, and *IR/iGluRs* in *P. saucia.*

| **ID** | **Forward primer (5'to 3')** | **Reverse primer (5' to 3')** |
| --- | --- | --- |
| ***ORs*** |  |  |
| *PsauORco* | ACTTACAGACCGAATACTGCT | GTGTCATACCGAAATCTTGCT |
| *PsauOR1* | ATCGATAATTGTTTCCCGTCA | TGAAACCCGTAATAAGCGAAC |
| *PsauOR3* | GTTCAACTTGATACCATTGCAC | TCGTTTCGTAATTCCACGGAT |
| *PsauOR4* | GGCCCTATTTACTTTTAACTGG | TTTAAAATACGCATGTGACCC |
| *PsauOR5* | TGTATTCTTTACCACCGGGAG | GAACATTCCAGCAATAACAGG |
| *PsauOR6* | GCCTGCTAATAAAGATATCGT | CGATGGTAATCTATGCACT |
| *PsauOR7* | GGCCCTGTTTAACATTAACCC | CCAATTTATAATGCAACCGACCAC |
| *PsauOR8* | TTTGAATGAAATGCACTTGCT | TATTGAACATGGCAAGACCAG |
| *PsauOR10* | CAATCAGACCCCTCACGTT | AAAATAAAACACATGGCCGAT |
| *PsauOR11* | CGCTTACTATGCTATGTACCG | TTCGACCCTTCAAATAGCTCT |
| *PsauOR12* | CGTGTACAATTCAGACTGCT | ATGCCATTTCACTAATTCTGC |
| *PsauOR13* | CGTTCACAGACATCATGCGTT | ACTCCTCGATCAACACGCAAC |
| *PsauOR14* | ACAACTTGAGCATTTTCGGCTA | ACGAATGTCATGATTCCGAT |
| *PsauOR15* | GCAATAAAGATAAGGACCGAT | TATCTCTAAAGTCCGCACGA |
| *PsauOR16* | CGGTCCAATACTTACTATCACC | CGAAGACATAAATCGTCCAGT |
| *PsauOR17* | AATTTTCTGTTCCTGTCGGAG | TTCACCACGTAAAGGATCGAG |
| *PsauOR18* | TTTCAGCTACAAGACGGAA | AGCTCGATATCTATCACCT |
| *PsauOR19* | TACAAGACTCCAGCATACGAA | GCCAGTATTTCTATTTGTCCGAT |
| *PsauOR20* | GGATTTTATCAGTAACATCGGTA | AAAATTAAGCAAAGCGTGAC |
| *PsauOR21* | CGGGAACTTATAAATCTCGT | GTCATTTGTTCTGTAACCGTA |
| *PsauOR22* | TGGCATCCTGTATTATATGTGT | TCTTGTCCATAATCCGTGTG |
| *PsauOR23* | TGTGATGAAACCTGTCGCCAT | ATGAAGAAGACTCCAGCGAT |
| *PsauOR24* | TGATATTGATTGCCATGCTTC | CATACAGTACATAAGCACCAC |
| *PsauOR25* | TACATCCAGCCACTAGCAA | AGTTTCTTGTAGACAATCGC |
| *PsauOR26* | AGCTGAGCAAAATATTCGGTT | CAATCGTTTCAGCGACACC |
| *PsauOR27* | GCTAGAACTACTTTTGCCAT | CAGCACATATATCGACGACCA |
| *PsauOR28* | CGGGTCATCGTCTACTTCACCT | CAAGTCCTCGAAGTACCCCTG |
| *PsauOR29* | CAAGCAAATGTTTCGGTCGT | ATCTCGAATACTTGATCATCGC |
| *PsauOR30* | CGCATCAGTAAACTATTCGT | GCGATCCACTAACTTGTGT |
| *PsauOR31* | AAAGATATAGCAAAACGCCAT | GTTCTGCTATAAGTGCGACCA |
| *PsauOR32* | CTTATTTTCGTGGTAACCCAA | CCTTGACTACACAGACCGTA |
| *PsauOR33* | CTCACAAACATTACGAGGA | CGTAATCATTTAAATAATCCGAA |
| *PsauOR34* | TCACCTAACTACGAGATCGCAT | TTTGTGACTCGGCGTACCCAA |
| *PsauOR35* | AACGTGTTCACAAAATACTGC | TACGTTCGCCCTATAAGTCTC |
| *PsauOR36* | ACTACGACAAGATTACCGGGACT | ACGCTCAGCACATTCAAACTGAC |
| *PsauOR37* | ATTTCAGATTCTACGCCAT | GTCAGAATTCCGATTCCCAA |
| *PsauOR38* | GAAATACATATCGTCGGCTTC | CTGCCCATAATTATATGAACGAA |
| *PsauOR39* | ACAAATACAGATGCTTCGCTAC | GTTTTCGCTTTGTCTTCGTCT |
| *PsauOR40* | GCAGATGATCAAACGGTCCA | TGAATGTTTCTCCCTTCCGTA |
| *PsauOR41* | TACTGATACGCTTAGCCCAA | GGCCATATTGACTATACCAT |
| *PsauOR42* | TATTCACCTTCCTGATGCTCGT | CTGACTAAGCCTCCTATCGCTA |
| *PsauOR43* | CTCTTCCGATTTACTATGCC | CTCTTTTCAATCAGCCGAGA |
| *PsauOR44* | TTCGAGCTACCCATTCAACCC | GTCATGATCAATGTTGCTCCC |
| *PsauOR45* | TCAAATTGCCCTTTCAGACGA | GTTATTTCCCCGGCGTTGCAT |
| *PsauOR47* | TTTAAAACTTCACGGAGCCAT | CGTAAGCATAAACACGGCAAA |
| *PsauOR48* | TCCGATAGAAACTCAAGACGA | TTATCATAGATGCCACGCCAA |
| *PsauOR49* | ACAGAACTACAATAAGCTCGT | TCATTAATGCGGACATGCAA |
| *PsauOR50* | AAGAACCATAGAGGACACGAA | AAAGTTAGCTCATATGCCACT |
| *PsauOR51* | CAGCCAAGATCGTGAACATCC | ATCATTTCCCGGTTACTGCTC |
| *PsauOR52* | AACACCGTTCGAGTTCCCAT | CGTAGTGTTACCGATCGCAACC |
| *PsauOR53* | GTGCCTTTCTCTTCATATCCG | CTGCGTCAAAGACATATTGGA |
| *PsauOR54* | TTCGTGGACCGCATAAAGCA | AAACTCTAGGCTCACTTCACC |
| *PsauOR55* | ATCATCGGTGAACCAAGACTG | TAGACAAATCCGGCGTACACA |
| *PsauOR56* | CCTAATGGAAGATTATGCGAT | TATAGCTTGAAACTCGCCTT |
| *PsauOR57* | TGGCATACTTAGTCATTGCAC | TCCATGTTGATTCATAGGCTG |
| *PsauOR58* | CCGTGAATTTAAATCAAAGCC | AATAACCACTAGGTACACGAA |
| *PsauOR59* | ATAATAAGAACACACGGAAGG | AATAGTTCCGCTATAAGACCA |
| *PsauOR60* | TCTTACTCCTCGGTGCCCAA | ATACATCAACTCGTAGGCCAT |
| *PsauOR61* | GCTTCTTCAATATTTCGGTGT | CTTCCCGTTTCGATTATCAGA |
| *PsauOR63* | GCACTACAACAAGATCACCGAGA | GCGAACTCGATGCTCAACACA |
| *PsauOR64* | GCAGTCTCATTTGACGCAGA | CGCCAGGTTGTACATCACCA |
| *PsauOR65* | CTATCAGGCCACTACGACCTT | TCTCCTCACACCAGCGACA |
| *PsauOR66* | AAAACATAGCAAAGAACCGAA | TTTGATGTCTTGAATCGTCCC |
| ***GRs*** |  |  |
| *PsauGR1* | TGATACTCATATCGACGTTGCT | CTCCACAACCACGATCATGC |
| *PsauGR2* | CGATAAATGCTGTCGCTCCAAAG | TCGTTATTGGCAAGACTCCCAT |
| *PsauGR3* | CCAGCAATCTGTTCTTCGTCT | ACAAAGTAGATCGAAGCCTCGT |
| *PsauGR4* | TACCTGTGGCTGAACTTGTCC | GGCTCCATAAACAGCAATCGTT |
| *PsauGR5* | CATATCCTTTGCCAATAACCTGT | TTCATGCCCGCCTAGTAGACC |
| *PsauGR6* | CCATTTCCTTAGTGTGGGCTA | TGTCATAAAGCCAACCACGAA |
| *PsauGR7* | CGTTAGACTTGATAGCTCACGTA | GTACACAATCCGATAACTGTCC |
| *PsauGR8* | TTAGCTGCCTCAGATGTCC | TGCCCGTTAAAGCCACGAA |
| *PsauGR9* | TATTCAGACTGGTACGCCCTT | AGGCCCATACTGATTAGCAC |
| *PsauGR10* | CTCTACTCTCTATGGTCGCTA | CCAGCCATATAAGTATGAGTTGC |
| ***IR/iGluRs*** |  |  |
| *PsauIR1.1* | CTACTACAGTGGCTTCACGAAC | TTCGACGCCTGGTTTAACAGT |
| *PsauIR1.2* | AAGACTGTGCTGTATCCTGT | ACTTCAAATGATGCTCTGCGTA |
| *PsauIR2* | ACGCCATCACTATTTACAAGG | TGTGATCGCCCAGTATCAGC |
| *PsauIR7d.1* | TTGAACATTTACCTGCGGAT | CGGCTCAATGTTAAAAGCTCT |
| *PsauIR7d.3* | GCAACTTCTACCGATGTCC | TACTTTGCCATCGTAACCAC |
| *PsauIR8a* | GAGATTACACTGAACGCAAC | GTTCTGAAGCGTTTATAGCC |
| *PsauIR21a* | CATCGCTTGTGAATTTACTCC | CCTTTAAGAAGATTTTCGACCA |
| *PsauIR25a* | AGAATCGTCACAGTCGAGCAA | CCGGAGCCAAAGATATTTCGT |
| *PsauIR40a* | CAAGTATTTCAAGCAACGGAAC | TCTGCCAGAGTCATGAACGA |
| *PsauIR41a* | ACGATAATTTCTGATGCTCCT | CGCACTATGTAATCAGAACACC |
| *PsauIR60a* | TTACGTATTTAGCCGATGCTT | ATAATTTTCCACGTTATACCAC |
| *PsauIR60a1b* | TAATGCTTCTTCGGACGGAT | TCCAAATAATGAAGACGCACT |
| *PsauIR64a* | ATTAAGTCCCAGATTGACGTG | GTATCCCCATGTACTGACTCG |
| *PsauIR68a* | GTACACTTCGCAATGTTGGAC | TCCCCGTTATCAGTAACACA |
| *PsauIR75d* | CAATCGGCAGATCGACACGTT | CGACACGCCGTACACGTCCA |
| *PsauIR75p.1* | TAATCAAAGCAGGGAACCACT | CCTTTCGGATTCTTTCAACACC |
| *PsauIR75p.2* | TTAATCAAAGCAGGGAACCAC | CCTTTCGGATTCTTTCAACACC |
| *PsauIR75p.3* | CTGAACTCGCCTCTCAAGCTG | GTTTCTTTCCTTTGGGTGCTA |
| *PsauIR75q.1* | AAGTATCTGGGAATTTCCGTA | CAAGTACCGTAGTATTCCGTA |
| *PsauIR75q.2* | AATTCTACGGAACTTGGAC | GTTAAATGATCGACGCTCT |
| *PsauIR76b* | ACGCTGTCCAAGTTTACCCTG | GTTGCTAATCATCGCGCTCA |
| *PsauIR85a* | AATTTTGAGAACTGACCGACT | TGAAGACACTTAAGCGTAGCC |
| *PsauIR87a* | TCTTGCCAAGGCTATAGTGT | ACCACATCATTATATTCGTCGT |
| *PsauIR93a* | TAACATCGCAGATAGACGACGAA | ATTTCACCGGCAACTTAGACA |
| *PsauiGluR2* | TCGCTACAGCACATTCAGTCG | ACAGATTAACAACGGACCAGT |
| *PsauiGluR3* | TCACCAAGCCGTTTATGAACC | CAGCAAGCACGTACAACCAG |
| *PsauiGluR4* | ATCTTTGGACCTTCACAGCGTA | TTCGTCGTAAACCCAATCCCT |
| *PsauiGluR6* | TATCGGGATCCTGTACAAGCA | GATCAGCAGCATGTAATACCAC |
| *PsauiGluR7* | CATTTGTGCCAGAATATCACC | TGATAGAGCCCATTGTTAGCC |
| *PsauiGluR8* | ATCATGAACAATATGGGCGCTCT | CGCTCGCTTCTCAAGACCAC |
| ***PsauActin*** | TCATCACCATCGGAAACGAAC | GCGTACAAGTCCTTACGGAT |
