# Supplementary material for: Identification of Candidate Chemosensory Receptors in the Antennae of the Variegated Cutworm, Peridroma saucia Hübner, Based on a Transcriptome Analysis
Source: Front Physiol. 2020 Jan 31;11:39. doi: 10.3389/fphys.2020.00039 (PMC7005060; doi:10.3389/fphys.2020.00039)
Supplement: TABLE S2 — Evaluation of sequencing data of P. saucia samples. [file Table_2.docx]

Table S2: Evaluation of sequencing data of *P. saucia* samples.

| Sample | Read number | Base number | GC content | ≥Q30 (%) |
| --- | --- | --- | --- | --- |
| FA1 | 25,131,161 | 6,304,961,298 | 46.18% | 89.62% |
| FA2 | 31,405,996 | 7,854,648,156 | 46.24% | 89.17% |
| FA3 | 26,758,619 | 6,694,887,898 | 46.34% | 89.94% |
| MA1 | 27,668,536 | 6,913,510,346 | 46.72% | 90.36% |
| MA2 | 24,759,845 | 6,182,796,264 | 47.27% | 90.47% |
| MA3 | 24,769,554 | 6,179,933,050 | 47.09% | 90.68% |

FA: female antennae; MA: male antennae
