# Supplementary material for: Identification of Candidate Chemosensory Receptors in the Antennae of the Variegated Cutworm, Peridroma saucia Hübner, Based on a Transcriptome Analysis
Source: Front Physiol. 2020 Jan 31;11:39. doi: 10.3389/fphys.2020.00039 (PMC7005060; doi:10.3389/fphys.2020.00039)
Supplement: TABLE S3 — Details for candidate odorant receptors in P. saucia antennae. [file Table_3.docx]

**Supplementary Table S3.** Details for candidate odorant receptors in *P. saucia* antennae

| **Name** | **ID** | **ORF**  **(aa)** | **BLASTx best hit (GenBank accession/name/species)** | **Full length** | **Identity**  **(%)** | **TMD (No)** | **E-value** |
| --- | --- | --- | --- | --- | --- | --- | --- |
| *PsauORco* | Contig3 | 473 | BAG71415.1\|olfactory receptor-2 [*Mythimna separata*] | Yes | 99 | 7 | 0.00E + 00 |
| *PsauOR1* | Contig12 | 443 | BAG71414.1\|olfactory receptor-1 [*Mythimna separata*] | Yes | 81 | 5 | 0.00E + 00 |
| *PsauOR3* | Contig7 | 435 | AGS41442.1\|olfactory receptor 3 [*Agrotis segetum*] | Yes | 91 | 5 | 0.00E + 00 |
| *PsauOR4* | Contig18 | 425 | BAG71423.2\|olfactory receptor [*Mythimna separata*] | Yes | 75 | 5 | 0.00E + 00 |
| *PsauOR5* | Contig13 | 432 | AGY14579.2\|putative odorant receptor [*Sesamia inferens*] | Yes | 84 | 4 | 0.00E + 00 |
| *PsauOR6* | Contig14 | 434 | AGI96751.1\|olfactory receptor 16 [*Spodoptera litura*] | Yes | 55 | 7 | 2.00E-167 |
| *PsauOR7* | Contig16 | 436 | AGI96751.1\|olfactory receptor 16 [*Spodoptera litura*] | Yes | 53 | 7 | 3.00E-156 |
| *PsauOR8* | Contig10 | 426 | AGY14579.2\|putative odorant receptor [*Sesamia inferens*] | Yes | 52 | 5 | 4.00E-161 |
| *PsauOR10* | Contig66 | 452 | AIG51891.1\|odorant receptor, partial [Helicoverpa armigera] | yes | 79 | 7 | 0.00E + 00 |
| *PsauOR11* | Contig4 | 458 | AIG51892.1\|odorant receptor [*Helicoverpa armigera*] | Yes | 81 | 7 | 0.00E + 00 |
| *PsauOR12* | Contig5 | 429 | AOE48017.1\|putative odorant receptor OR12 [*Athetis lepigone*] | Yes | 84 | 7 | 0.00E + 00 |
| *PsauOR13* | Contig6 | 455 | ABQ84982.1\|putative chemosensory receptor 12 [*Spodoptera littoralis*] | Yes | 86 | 7 | 0.00E + 00 |
| *PsauOR14* | Contig8 | 423 | AOE48025.1\|putative odorant receptor OR20 [*Athetis lepigone*] | Yes | 76 | 7 | 0.00E + 00 |
| *PsauOR15* | Contig9 | 424 | AOE48019.1\|putative odorant receptor OR14 [*Athetis lepigone*] | No | 75 | 6 | 0.00E + 00 |
| *PsauOR16* | Contig11 | 445 | ALM26230.1\|odorant receptor 40 [*Athetis dissimilis*] | Yes | 67 | 7 | 0.00E + 00 |
| *PsauOR17* | Contig15 | 392 | ALM26209.1\|odorant receptor 20 [*Athetis dissimilis*] | Yes | 80 | 7 | 0.00E + 00 |
| *PsauOR18* | Contig17 | 422 | BAR43488.1\|putative olfactory receptor 46 [*Ostrinia furnacalis*] | Yes | 50 | 7 | 9.00E-148 |
| *PsauOR19* | Contig19 | 429 | AOE48055.1\|putative odorant receptor OR50 [*Athetis lepigone*] | Yes | 93 | 7 | 0.00E + 00 |
| *PsauOR20* | Contig20 | 411 | ALM26206.1\|odorant receptor 17 [*Athetis dissimilis*] | Yes | 74 | 7 | 0.00E + 00 |
| *PsauOR21* | Contig21 | 400 | AIG51899.1\|odorant receptor [*Helicoverpa armigera*] | Yes | 64 | 7 | 0.00E + 00 |
| *PsauOR22* | Contig22 | 394 | ALM26196.1\|odorant receptor 8 [*Athetis dissimilis*] | Yes | 75 | 7 | 0.00E + 00 |
| *PsauOR23* | Contig23 | 420 | ALM26228.1\|odorant receptor 38 [*Athetis dissimilis*] | Yes | 88 | 7 | 0.00E + 00 |
| *PsauOR24* | Contig24 | 413 | ALM26235.1\|odorant receptor 45 [*Athetis dissimilis*] | Yes | 75 | 7 | 0.00E + 00 |
| *PsauOR25* | Contig25 | 420 | ALM26211.1\|odorant receptor 22 [*Athetis dissimilis*] | Yes | 67 | 7 | 1.00E-158 |
| *PsauOR26* | Contig26 | 408 | AIG51882.1\|odorant receptor [*Helicoverpa armigera*] | Yes | 76 | 7 | 1.00E-162 |
| *PsauOR27* | Contig27 | 408 | AIG51860.1\|odorant receptor [*Helicoverpa armigera*] | Yes | 82 | 7 | 0.00E + 00 |
| *PsauOR28* | Contig28 | 412 | AOE48060.1\|putative odorant receptor OR55 [*Athetis lepigone*] | Yes | 72 | 6 | 0.00E + 00 |
| *PsauOR29* | Contig29 | 397 | AIG51879.1\|odorant receptor [*Helicoverpa armigera*] | Yes | 84 | 5 | 0.00E + 00 |
| *PsauOR30* | Contig30 | 395 | ALM26219.1\|odorant receptor 30 [*Athetis dissimilis*] | Yes | 84 | 5 | 0.00E + 00 |
| *PsauOR31* | Contig31 | 407 | KOB74670.1\|odorant receptor 50 [*Operophtera brumata*] | No | 58 | 6 | 3.00E-175 |
| *PsauOR32* | Contig32 | 406 | AOE48046.1\|putative odorant receptor OR41 [*Athetis lepigone*] | Yes | 78 | 6 | 0.00E + 00 |
| *PsauOR33* | Contig33 | 400 | AIZ00994.1\|putative olfactory receptor 9 [*Helicoverpa armigera*] | Yes | 71 | 6 | 0.00E + 00 |
| *PsauOR34* | Contig34 | 407 | ALM26227.1\|odorant receptor 37 [*Athetis dissimilis*] | No | 68 | 4 | 0.00E + 00 |
| *PsauOR35* | Contig35 | 402 | AIG51887.1\|odorant receptor [*Helicoverpa armigera*] | Yes | 89 | 6 | 0.00E + 00 |
| *PsauOR36* | Contig36 | 390 | AIG51873.1\|odorant receptor [*Helicoverpa armigera*] | Yes | 85 | 4 | 0.00E + 00 |
| *PsauOR37* | Contig37 | 387 | AOE48027.1\|putative odorant receptor OR22 [*Athetis lepigone*] | Yes | 79 | 5 | 0.00E + 00 |
| *PsauOR38* | Contig38 | 404 | AOE48066.1\|putative odorant receptor OR61 [*Athetis lepigone*] | Yes | 83 | 4 | 0.00E + 00 |
| *PsauOR39* | Contig39 | 406 | AOE48024.1\|putative odorant receptor OR19 [*Athetis lepigone*] | Yes | 85 | 4 | 0.00E + 00 |
| *PsauOR40* | Contig40 | 392 | AOE48037.1\|putative odorant receptor OR32 [*Athetis lepigone*] | No | 76 | 6 | 0.00E + 00 |
| *PsauOR41* | Contig41 | 395 | ALM26250.1\|odorant receptor 85 [*Athetis dissimilis*] | Yes | 75 | 6 | 0.00E + 00 |
| *PsauOR42* | Contig42 | 402 | ALM26208.1\|odorant receptor 19 [*Athetis dissimilis*] | Yes | 71 | 6 | 0.00E + 00 |
| *PsauOR43* | Contig43 | 395 | ALM26245.1\|odorant receptor 62 [*Athetis dissimilis*] | Yes | 91 | 6 | 0.00E + 00 |
| *PsauOR44* | Contig44 | 406 | ALM26230.1\|odorant receptor 40 [*Athetis dissimilis*] | Yes | 74 | 5 | 0.00E + 00 |
| *PsauOR45* | Contig45 | 401 | ALM26210.1\|odorant receptor 21 [*Athetis dissimilis*] | Yes | 80 | 6 | 0.00E + 00 |
| *PsauOR47* | Contig47 | 400 | ACL81185.1\|putative olfactory receptor 18 [*Agrotis segetum*] | Yes | 96 | 5 | 0.00E + 00 |
| *PsauOR48* | Contig48 | 390 | ALM26204.1\|odorant receptor 15 [*Athetis dissimilis*] | Yes | 66 | 7 | 0.00E + 00 |
| *PsauOR49* | Contig49 | 385 | ALM26205.1\|odorant receptor 16 [*Athetis dissimilis*] | Yes | 89 | 5 | 0.00E + 00 |
| *PsauOR50* | Contig50 | 392 | AIG51902.1\|odorant receptor [*Helicoverpa armigera*] | Yes | 75 | 3 | 0.00E + 00 |
| *PsauOR51* | Contig51 | 392 | AOE48058.1\|putative odorant receptor OR53 [*Athetis lepigone*] | Yes | 85 | 7 | 0.00E + 00 |
| *PsauOR52* | Contig52 | 393 | AOE48064.1\|putative odorant receptor OR59 [*Athetis lepigone*] | Yes | 80 | 6 | 0.00E + 00 |
| *PsauOR53* | Contig53 | 390 | AJG42376.1\|olfactory receptor 10 [*Helicoverpa armigera*] | Yes | 94 | 5 | 0.00E + 00 |
| *PsauOR54* | Contig54 | 387 | AOE48014.1\|putative odorant receptor OR9 [*Athetis lepigone*] | No | 64 | 4 | 0.00E + 00 |
| *PsauOR55* | Contig55 | 390 | AIG51903.1\|odorant receptor, partial [*Helicoverpa armigera*] | Yes | 78 | 4 | 0.00E + 00 |
| *PsauOR56* | Contig56 | 388 | BAR43488.1\|putative olfactory receptor 46 [*Ostrinia furnacalis*] | No | 49 | 5 | 2.00E-132 |
| *PsauOR57* | Contig57 | 379 | AGK89999.1\|olfactory receptor 3 [*Helicoverpa armigera*] | Yes | 86 | 7 | 0.00E + 00 |
| *PsauOR58* | Contig58 | 381 | AOE48063.1\|putative odorant receptor OR58 [*Athetis lepigone*] | No | 80 | 4 | 0.00E + 00 |
| *PsauOR59* | Contig59 | 380 | KOB74670.1\|odorant receptor 50 [*Operophtera brumata*] | No | 63 | 6 | 5.00E-171 |
| *PsauOR60* | Contig60 | 356 | AII01092.1\|odorant receptor [*Dendrolimus kikuchii*] | No | 61 | 4 | 1.00E-161 |
| *PsauOR61* | Contig88 | 329 | AOE48041.1\|putative odorant receptor OR36 [*Athetis lepigone*] | No | 82 | 6 | 0.00E + 00 |
| *PsauOR63* | Contig63 | 316 | AOE48064.1\|putative odorant receptor OR59 [*Athetis lepigon*e] | No | 84 | 3 | 8.00E-131 |
| *PsauOR64* | Contig64 | 258 | ALM26217.1\|odorant receptor 28 [*Athetis dissimilis*] | No | 72 | 4 | 9.00E-138 |
| *PsauOR65* | Contig65 | 236 | AOE48030.1\|putative odorant receptor OR25 [*Athetis lepigone*] | No | 88 | 3 | 4.00E-164 |
| *PsauOR66* | Contig2 | 442 | AOE48019.1\|putative odorant receptor OR14 [*Athetis lepigone*] | Yes | 88 | 6 | 0.00E + 00 |
