# Supplementary material for: Identification of Candidate Chemosensory Receptors in the Antennae of the Variegated Cutworm, Peridroma saucia Hübner, Based on a Transcriptome Analysis
Source: Front Physiol. 2020 Jan 31;11:39. doi: 10.3389/fphys.2020.00039 (PMC7005060; doi:10.3389/fphys.2020.00039)
Supplement: TABLE S4 — Details for candidate gustatory receptors in P. saucia antennae. [file Table_4.docx]

**Table S4.** Details for candidate gustatory receptors in *P. saucia* antennae.

| **Name** | **ID** | **ORF**  **(aa)** | **BLASTx best hit (GenBank accession/name/species)** | **Full length** | **Identity**  **(%)** | **TMD (No)** | **E-value** |
| --- | --- | --- | --- | --- | --- | --- | --- |
| *PsauGR1* | Contig395 | 512 | AGA04648.1\|gustatory receptor [*Helicoverpa armigera*] | Yes | 90 | 6 | 0.00E + 00 |
| *PsauGR2* | Contig396 | 475 | AMZ01262.1\|gustatory receptor [*Helicoverpa armigera*] | Yes | 90 | 6 | 0.00E + 00 |
| *PsauGR3* | Contig398 | 476 | AIG51911.1\|gustatory receptor [*Helicoverpa armigera*] | No | 79 | 6 | 0.00E + 00 |
| *PsauGR4* | Contig399 | 433 | AIG51908.1\|gustatory receptor [*Helicoverpa armigera*] | Yes | 96 | 7 | 0.00E + 00 |
| *PsauGR5* | Contig108 | 453 | AGK90010.1\|gustatory receptor 1 [*Helicoverpa armigera*] | Yes | 91 | 8 | 0.00E + 00 |
| *PsauGR6* | Contig118 | 427 | AGK90012.1\|gustatory receptor 5 [*Helicoverpa armigera*] | Yes | 80 | 6 | 0.00E + 00 |
| *PsauGR7* | Contig156 | 414 | ALM26258.1\|gustatory receptor 10, partial [*Athetis dissimilis*] | Yes | 69 | 7 | 2.00E-103 |
| *PsauGR8* | Contig128 | 402 | ALM26257.1\|gustatory receptor 8, partial [*Athetis dissimilis*] | Yes | 81 | 6 | 0.00E + 00 |
| *PsauGR9* | Contig148 | 355 | ALM26256.1\|gustatory receptor 7, partial [*Athetis dissimilis*] | No | 85 | 6 | 4.00E-176 |
| *PsauGR10* | Contig138 | 355 | ALS03936.1\|gustatory receptor 1 [*Ectropis obliqua*] | Yes | 85 | 7 | 1.00E-133 |
