# Supplementary material for: Identification of Candidate Chemosensory Receptors in the Antennae of the Variegated Cutworm, Peridroma saucia Hübner, Based on a Transcriptome Analysis
Source: Front Physiol. 2020 Jan 31;11:39. doi: 10.3389/fphys.2020.00039 (PMC7005060; doi:10.3389/fphys.2020.00039)
Supplement: TABLE S5 — Details for candidate IRs/iGluRs in P. saucia antennae. [file Table_5.docx]

**Table S5.** Details for candidate IRs/iGluRs in *P. saucia* antennae.

| **Name** | **ID** | **ORF**  **(aa)** | **BLASTx best hit (GenBank accession/name/species)** | **Full length** | **Identity**  **(%)** | **TMD (No)** | **E-value** |
| --- | --- | --- | --- | --- | --- | --- | --- |
| *PsauIR1.1* | Contig296 | 652 | ADR64688.1\|putative chemosensory ionotropic receptor IR1 [*Spodoptera littoralis*] | Yes | 67% | 3 | 0.00E + 00 |
| *PsauIR1.2* | Contig341 | 569 | AOE48004.1\|putative ionotropic receptor IR1.2 [*Athetis* *lepigone*] | No | 80% | 3 | 0.00E + 00 |
| *PsauIR2* | Contig650 | 611 | AOG12849.1\|ionotropic receptor [*Eogystia hippophaecolus*] | Yes | 58% | 3 | 1.00E-44 |
| *PsauIR7d.1* | 59349_c0_g2_i1 | 595 | AOE47998.1\|putative ionotropic receptor IR7d.1 [*Athetis lepigone*] | Yes | 72% | 5 | 1.00E-17 |
| *PsauIR7d.3* | Contig379 | 585 | AOE48005.1\|putative ionotropic receptor IR7d.3 [*Athetis* *lepigone*] | Yes | 84% | 3 | 0.00E + 00 |
| *PsauIR8a* | 59826_c2_g1_i1 | 898 | ALM24945.1\|ionotropic receptor 8a.1 [*Athetis dissimilis*] | Yes | 91% | 4 | 0.00E + 00 |
| *PsauIR21a* | Contig106 | 856 | ALM24946.1\|ionotropic receptor 21a.3 [*Athetis dissimilis*] | Yes | 86% | 3 | 0.00E + 00 |
| *PsauIR25a* | 56817_c0_g2_i1 | 925 | AOE47999.1\|putative ionotropic receptor IR25a [*Athetis lepigone*] | Yes | 97% | 3 | 0.00E + 00 |
| *PsauIR40a* | 53738_c0_g1_i3 | 711 | AOE47989.1\|putative ionotropic receptor IR40a [*Athetis* *lepigone*] | Yes | 95% | 4 | 0.00E + 00 |
| *PsauIR41a* | 60814_c3_g15_i2 | 610 | AOE47997.1\|putative ionotropic receptor IR41a [*Athetis* *lepigone*] | Yes | 82% | 3 | 0.00E + 00 |
| *PsauIR60a* | 58935_c0_g1_i1 | 663 | KOB72397.1\|ionotropic receptor [*Operophtera brumata*] | Yes | 57% | 4 | 0.00E + 00 |
| *PsauIR60a1b* | 9428_c0_g1_i1 | 589 | AMM70717.1\|ionotropic receptor 60a1b, partial [*Heliconius clysonymus tabaconas*] | No | 39% | 3 | 1.00E-145 |
| *PsauIR64a* | Contig102 | 603 | BAR64801.1\|ionotropic receptor [*Ostrinia furnacalis*] | Yes | 49% | 3 | 2.00E-175 |
| *PsauIR68a* | Contig267 | 689 | AOE47990.1\|putative ionotropic receptor IR [*Athetis lepigone*] | Yes | 88% | 4 | 0.00E + 00 |
| *PsauIR75d* | Contig390 | 609 | ALM24944.1\|ionotropic receptor 75d [*Athetis dissimilis*] | Yes | 76% | 3 | 0.00E + 00 |
| *PsauIR75p.1* | 57923_c0_g1_i1 | 618 | ADR64684.1\|putative chemosensory ionotropic receptor IR75p [*Spodoptera littoralis*] | Yes | 68% | 3 | 0.00E + 00 |
| *PsauIR75p.2* | 57923_c0_g1_i2 | 622 | ADR64684.1\|putative chemosensory ionotropic receptor IR75p [*Spodoptera littoralis*] | Yes | 87% | 3 | 0.00E + 00 |
| *PsauIR75p.3* | Contig414 | 623 | AMM70652.1\|ionotropic receptor 75p [*Heliconius melpomene rosina*] | No | 71% | 2 | 0.00E + 00 |
| *PsauIR75q.1* | Contig449 | 635 | ADR64686.1\|putative chemosensory ionotropic receptor IR75q.1 [*Spodoptera littoralis*] | Yes | 55% | 3 | 0.00E + 00 |
| *PsauIR75q.2* | Contig450 | 625 | ALM24940.1\|ionotropic receptor 75q.2 [*Athetis dissimilis*] | Yes | 86% | 4 | 0.00E + 00 |
| *PsauIR76b* | Contig649 | 542 | AOE47987.1\|putative ionotropic receptor IR76b [Athetis lepigone] | yes | 90% | 3 | 0.00E + 00 |
| *PsauIR85a* | 52215_c0_g1_i1 | 621 | AMM70659.1\|ionotropic receptor 85a [*Heliconius* *melpomene rosina*] | Yes | 57% | 3 | 1.00E-26 |
| *PsauIR87a* | 55838_c0_g1_i1 | 644 | AOE47991.1\|putative ionotropic receptor IR87a, partial [*Athetis lepigone*] | Yes | 91% | 3 | 0.00E + 00 |
| *PsauIR93a* | Contig687 | 872 | BAR64811.1\|ionotropic receptor [*Ostrinia furnacalis*] | Yes | 78% | 3 | 0.00E + 00 |
| *PsauiGluR2* | 41691_c0_g1_i1 | 891 | AIG51925.1\|ionotropic glutamate receptor [*Helicoverpa armigera*] | No | 91% | 3 | 0.00E + 00 |
| *PsauiGluR3* | Contig542 | 890 | AIG51926.1\|ionotropic glutamate receptor, partial [*Helicoverpa armigera*] | Yes | 96% | 4 | 0.00E + 00 |
| *PsauiGluR4* | Contig283 | 864 | AIG51927.1\|ionotropic glutamate receptor [*Helicoverpa* *armigera*] | No | 99% | 3 | 0.00E + 00 |
| *PsauiGluR6* | Contig684 | 443 | AIG51929.1\|ionotropic glutamate receptor [*Helicoverpa* *armigera*] | No | 83% | 3 | 0.00E + 00 |
| *PsauiGluR7* | Contig48 | 936 | AIG51930.1\|ionotropic glutamate receptor [*Helicoverpa* *armigera*] | Yes | 77% | 3 | 0.00E + 00 |
| *PsauiGluR8* | 53530_c0_g4_i1 | 1058 | AIG51931.1\|ionotropic glutamate receptor, partial [*Helicoverpa armigera*] | Yes | 99% | 6 | 0.00E + 00 |
